# Clinical correlation between HBV infection and concomitant bacterial infections

**DOI:** 10.1038/srep15413

**Published:** 2015-12-04

**Authors:** Wei Li, Ronghua Jin, Peng Chen, Guoxian Zhao, Ning Li, Hao Wu

**Affiliations:** 1 Center for Infectious Diseases, Beijing You’an Hospital, Capital Medical University, Beijing 100069, China; 2 Department of Administration, Beijing You’an Hospital, Capital Medical University, Beijing 100069, China

## Abstract

Bacterial infections are common in patients suffering viral hepatitis and critical for prognosis. However, any correlation between HBV and concomitant bacterial infections is not well characterized. A retrospective study was conducted from Jan 2012 to Jan 2014 on 1333 hospitalized patients infected with bacteria. Among them, 491 HBV-infected patients were co-infected with *E. coli* (268)*, S. aureus* (61), *P. aeruginosa* (64) or *K. pneumoniae* (98). A group of 300 complication-free chronically HBV-infected patients were controls. We found that HBV DNA levels were elevated in patients with each of the bacterial infections (all P < 0.05). ALT and HBeAg were strong determinants of high HBV DNA concentration. Patterns of determinants varied in infections by Gram-positive and Gram-negative bacteria. Patients with HBV DNA ≥ 2000 IU/mL had higher rates of all four concomitant bacterial infections (all P < 0.001). All types of strains isolated from HBV-positive patients showed less resistance to tested antimicrobials. The HBV DNA serum concentrations were inversely correlated to the number of ineffective antimicrobials in *E. coli, P. aeruginosa* and *K. pneumoniae* infections (P = 0.022, 0.017 and 0.016, respectively), but not *S. aureus* (P = 0.194). In conclusion, bacterial infections are associated with a high level of HBV replication, which, in turn, has a significant positive impact on bacterial resistance to antimicrobials. These correlations vary between Gram-negative and Gram-positive bacteria.

Hepatitis B virus (HBV) is one of the leading infectious diseases in the world in terms of the number of sufferers and the clinical significance, particularly in China^1^. Even with currently recommended first-line treatment regimens, such as nucleos(t)ide analogs (NAs) and interferons, complications such as bacterial infections, ascites, spontaneous peritonitis, etc., may still occur. Many HBV-infected patients progress to late stage diseases such as cirrhosis and hepatocellular carcinoma (HCC)^2^,^3^. Use of pegylated interferon-alpha for 48 weeks can result in long-lasting control of the disease, but treatment failure is seen in the majority of patients^4^,^5^. Life-long use of NAs has been proven to result in sustained viral suppression, but its safety and efficacy are still unknown^6^,^7^,^8^, and it usually cannot clear the virus from infected cells, suggesting that the disease progression may not be stopped and the risk of development of significant complications is not eliminated. Novel treatment strategies are required.

Bacterial infections are commonly seen in patients with hepatitis, including HBV-induced hepatitis. For example, *Escherichia coli*, *Staphylococcus aureus*, *Pseudomonas aeruginosa* and *Klebsiella pneumoniae* are all common human pathogens in infections associated with ascites, a serious complication of end-stage liver disorders. Liver abscess are commonly caused by *S. aureus*, *P. aeruginosa* and, especially, *K. pneumoniae*^9^,^10^. Infections with a novel hypervirulent *K. pneumoniae* (hv*KP*) strain can even lead to liver abscess in healthy young adults^11^,^12^. *P. aeruginosa* is one of the most common causes of bacteremia in liver transplant recipients^13^. Recently, urinary tract *E. coli* infection has been reported to make a significant contribution to the development of primary biliary cirrhosis (PBS)^14^.

Although a previous study gave some hints about potential effects of HBV on bacteria *in vitro*^15^, the crosstalk between HBV infection and concomitant bacterial infections, and its significance, is not well characterized. In the present study, we involved 491 HBV-infected patients co-infected with *E. coli*, *S. aureus*, *P. aeruginosa* or *K. pneumoniae*, and analyzed potential correlations between HBV infection and different common concomitant bacterial infections.

## Materials and Methods

### Subjects

A retrospective study was conducted in Beijing You’an Hospital (Beijing, PR China) on consecutive hospitalized patients with a positive culture for *E. coli, S. aureus, P. aeruginosa* or *K. pneumoniae* from Jan 2012 to Jan 2014. Beijing You’an Hospital is a tertiary major hospital specializing in liver disorders and infectious diseases. A total of 1333 patients were identified through bacterial cultures. Patients infected with more than one type of bacteria were excluded. Patients were also excluded if they were co-infected with hepatitis C virus (HCV) or human immunodeficiency virus (HIV). Inclusion criteria for the present study were: detectable hepatitis B surface antigen (HBsAg), age ≥18 years, and at least one positive culture for *E. coli, S. aureus, P. aeruginosa* or *K. pneumoniae.* Culture samples included blood, urine, stool, catheter, abscess puncture fluids, ascites, sputum, and bile. All blood samples were drawn upon admission of the patients to the hospital and were sent to the central laboratory for blood tests (including HBV-related blood tests) and bacterial cultures. Clinical and laboratory data were gathered and analyzed. Demographic and clinical characteristics, including age, sex, fibrosis stage, alanine transaminase (ALT) and gamma-glutamyl transaminase (GGT) serum levels, body mass index (BMI), platelet count, the presence or absence of diabetes or liver cancer, and HBV-related markers such as HBV DNA concentration, HBsAg titer, HBeAg, etc., were extracted from clinical databases. Liver fibrosis was classified according to Ishak’s score. An age-matched, sex-matched and bacterial infection-free group of 300 chronically HBV-infected patients were selected as controls.

### Ethics Statement

Beijing You’an Hospital Ethics Committee has approved this study and all relevant experiments. Approval covered the retrospective analysis of all patients and control subjects. All subjects gave written informed consent upon admission for their information to be stored and used for research. All experiments were performed in accordance with the human experimentation guidelines of the PR China, which were followed in the conduct of this clinical research.

### Clinical microbiological characterization of the bacterial strains

The 618 (268 isolated from HBV(+) patients) *E. coli*, 210 (61 isolated from HBV(+) patients) *S. aureus*, 256 (64 isolated from HBV(+) patients) *P. aeruginosa*, and 249 (98 isolated from HBV(+) patients) *K. pneumoniae* strains had been frozen and stored at −80 °C. Susceptibility testing (Phoenix 100 automated microbiology system (BD, NJ, USA)) to amikacin, amoxicillin-clavulanate, ampicillin, ampicillin-sulbactam, aztreonam, ceftizoxime, cefepime, cefotaxime, ceftazidime, ciprofloxacin, gentamicin, levofloxacin, piperacillin, tetracycline, SMZ-TMP, chloramphenicol, imipenem, meropenem and piperacillin-tazobactam was performed on each Gram-negative strain (*E. coli, P. aeruginosa,* and *K. pneumoniae*). Extended spectrum β-lactamase (ESBL) production was also determined by the Phoenix 100 system. Susceptibility testing to amoxicillin, ciprofloxacin, clindamycin, gentamicin, linezolid, oxacillin, penicillin, dalfopristin, rifampicin, tetracycline, Trimethoprim-sulfamethoxazole (SMZ-TMP), ampicillin, vancomycin, erythromycin, amikacin, furadantin, tobramycin, teicoplanin, and trimethoprim was preformed on every Gram-positive strain (*S. aureus*).

### Quantification of HBV DNA and HBsAg serum levels

Serum HBV DNA levels were quantified by a COBAS Amplicor HBV Monitor (Roche Molecular Systems, Pleasanton, CA, USA) with a detection limit of 20 IU/mL. HBsAg titer was determined with the Architect HBs-Antigen QT assay (Abbott Laboratories, Wiesbaden, Germany) based on an automated chemiluminescent microparticle immunoassay, following the manufacturer’s recommendations. The Architect HBs-Antigen QT assay measures a range of HBsAg from 0.05 to 250 IU/mL. Samples with higher HBsAg titer required dilution to bring them into the range of the calibration curve.

### Statistical analysis

SPSS software (version 15.0) was used for data analysis. Student’s *t*-test and the Wilcoxon rank-sum test were used for analysis of continuous variables. Continuous variables were assessed for normality and are presented as the mean ± SEM. Continuous variables were compared with Spearman’s rho correlation analysis. Categorical variables were compared with the chi-square test or Fisher’s exact test. P values of 0.05/n (n = the number of comparisons) are considered statistically significant based on the Bonferroni correction for multiple comparisons. A statistical trend was defined as a P value < 0.1 but >0.05/n. Logistic regression was used to analyze risk factors for HBV DNA concentration. All variables with a P value < 0.05 were included in the multivariate model. Forward selection with use of the likelihood-ratio test was used to select the final multivariate model for determinants of HBV DNA concentration.

## Results

### Patient characteristics

A total of 491 patients were selected for the current study according to the criteria described above. Baseline characteristics of these patients are summarized in Table 1. Another 300 age- and sex-matched chronically HBV-infected (CHB) controls without bacterial infections were involved as controls. Most of the patients were male (n = 395; 80.4%). HBV-infected patients without bacterial co-infection, were randomly selected from 497 patients in the same study period (n = 415, 83.5%, P = 0.598). The majority of the HBV-infected patients were HBeAg negative (n = 310; 63.1%).

### Correlation between HBV DNA serum levels and different bacterial co-infections

Mean serum HBV DNA levels in patients with each of the four different bacterial infections showed a significant elevation if compared to those in CHB patients without bacterial infections (*E. coli* (P = 0.002), *P. aeruginosa* (P = 0.008), *K. pneumoniae* (P = 0.003), *S. aureus* (P = 0.010)). Therefore, we performed uni- and multivariate logistic regression analysis of potential determinants of HBV DNA levels in patients with different co-infections. In all four bacterial infections, ALT and HBeAg were strong determinants of high HBV DNA concentration in both uni- and multivariate analysis. Liver cancer was significant only for Gram-negative bacterial infections (*P. aeruginosa* and *K. pneumoniae*) in both uni- and multivariate analysis. The combinations of determinants of HBV DNA concentration were almost the same in patients co-infected with HBV and *E. coli* or *K. pneumoniae*, where fibrosis, ALT, BMI and HBeAg were independent determinants. Liver cancer was an independent determinant in *K. pneumoniae* infection (P = 0.048) but not in *E. coli* infection (P = 0.071). Meanwhile, in patients co-infected with *S. aureus*, only ALT and HBeAg were independently correlated to serum HBV DNA levels. HBsAg serum level was not associated with HBV DNA concentration in patients with any bacterial infection (Table 2). Furthermore, we stratified patients according to HBV DNA serum level into two groups (<2000 versus ≥2000 IU/mL). This viral load threshold is generally considered a critical cut-off point for clinical decision making. We found that patients with HBV DNA ≥ 2000 IU/mL had substantially higher rates of all four concomitant bacterial infections, compared to those with HBV DNA < 2000 IU/mL (all four P values < 0.001).

### Strains isolated from patients with HBV infection showed less resistance to antimicrobials

**Figure 1:**
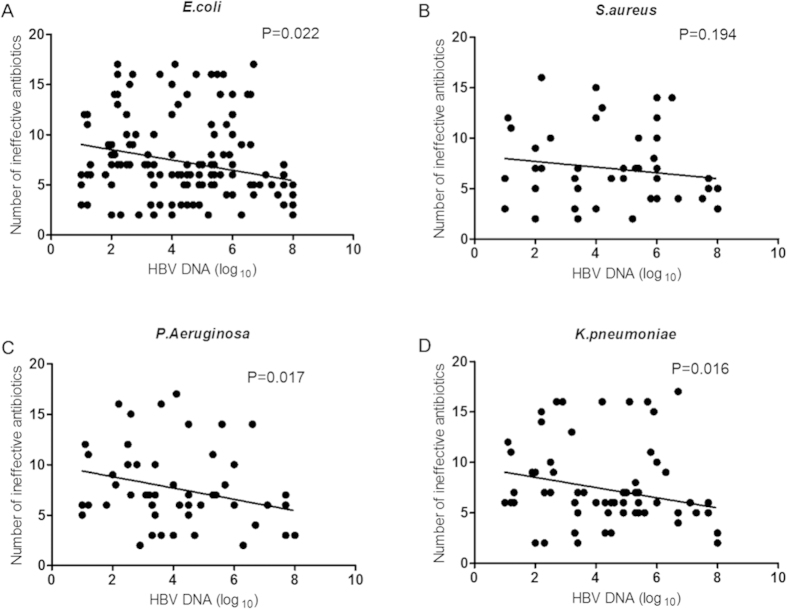
Correlation between serum HBV-DNA concentration and different bacterial infections.

To compare the changes in drug resistance of strains from patients with or without HBV infection, we collected all strains isolated from HBV(−) patients admitted in the same study period and compared their resistance to 19 antimicrobials with that of strains isolated from HBV(+) patients (Table 3). All four kinds of bacterial strains isolated from HBV(+) patients showed less resistance to the tested antimicrobials compared to those from HBV(−) patients. In HBV(+) patients, *E. coli* strains showed less resistance to 13 antibiotics compared to isolates from HBV(−) patients. *P. aeruginosa* strains were less resistant to 11 antibiotics, *K. pneumoniae* strains to 13, and *S. aureus* strains to 11. All Gram-negative strains were sensitive to carbapenems (imipenem and meropenem) and chloramphenicol, and resistant to ampicillin. No difference in resistance to piperacillin was observed between strains isolated from patients with or without HBV infections. The percentages of strains expressing extended spectrum β-lactamase (ESBL) among strains isolated from HBV(+) patients and HBV(−) patients were compared. The rates were lower in all three types of Gram-negative isolates from HBV(+) patients compared to the isolates from HBV(−) patients (P = 0.004, 0.009 and 0.007 for *E. coli, P. aeruginosa* and *K. pneumoniae*, respectively) (Table 3). The HBV DNA serum concentrations in HBV(+) patients with concomitant bacterial infection were inversely correlated to the number of ineffective antimicrobials in patients with *E. coli*, *P. aeruginosa* and *K. pneumoniae* infections (P = 0.022, 0.017 and 0.016, respectively), but this was not the case for *S. aureus* infection (P = 0.194) (Fig. 1). HBeAg positive status was also found to be inversely associated with the number of ineffective antimicrobials in patients with *E. coli*, *P. aeruginosa* and *K. pneumoniae* infections (P = 0.043, 0.025 and 0.047, respectively), but not *S. aureus* (P = 0.228).

## Discussion

In the present study, we investigated the crosstalk between HBV infection and common concomitant bacterial infections in 491 patients with HBV and bacterial co-infections. A previous study indicated that a peptide extracted from HBV demonstrated potent antimicrobial activity *in vitro*, leading to the possibility that high HBV DNA concentration may contribute to control of bacterial infection^15^. However, we found significant elevation of mean serum HBV DNA concentrations in patients with bacterial co-infections compared to that in controls. Possible explanations for this correlation could be that it is the high HBV DNA levels that lead to an increased chance of bacterial infections, or that the bacterial infections caused an elevation of serum HBV DNA, or both. Additional investigations are needed to distinguish between these possibilities.

In our study, all four kinds of bacterial strains isolated from HBV(+) patients showed less resistance to the antimicrobials we tested compared to bacteria isolated from HBV(−) patients. The resistance to antimicrobials appeared to be inversely correlated to HBV DNA serum concentration. The reason for this correlation remains unknown. Direct evidence on the crosstalk between HBV and bacterial infections is very rare in previous studies. However, both viral and bacterial infections are controlled by the host immune system. HBV infection has a great impact on the immune system and may compromise its ability to contain concomitant bacterial infections, leading to less chance of emerging new resistance to antimicrobials. Further studies are necessary to confirm this hypothesis. Patients infected with *S. aureus* strains showed differences from those infected with the other bacteria in terms of the correlation between HBV DNA serum concentration and the number of ineffective antimicrobials (Fig. 1). Antimicrobials against different types of bacteria have different mechanisms, which may contribute to this difference. In addition, bacteria that Gram-stain differently share little membrane structure and few antigens; their stimulation of and interaction with the host immune system and antimicrobials may vary significantly. In Table 2, we also observed different combinations of independent determinants of HBV DNA levels in patients infected with *S. aureus* from those in patients infected with the other three bacteria. This difference between *S. aureus* and the other three bacteria can probably be explained by their potentially different impacts on the host immune system, which in turn may affect HBV DNA levels and the emergence of resistance to antimicrobials. This observation and hypothesis may provide the basis for further studies; however, the underlying mechanisms remain unclear.

Our study has several limitations. First, clinical association studies cannot indicate a causal relationship. Therefore, the regulatory relationship between HBV infection and concomitant bacterial infections remains to be revealed. Our study provides the basis for the design of further *in vitro* investigation, or interventional clinical trials. Second, the study population in this work is not representative for all phases of HBV infection. All patients involved were admitted to hospital with significant clinical conditions, and those with mild chronic HBV infections are underrepresented. Third, the numbers of HBV-infected patients co-infected with each bacterium are not large enough to draw final conclusions, especially for the HBeAg negative patients. Larger studies are required to fully address all questions in this study.

In summary, we found that higher HBV DNA serum levels were observed in patients with concomitant bacterial co-infections if compared to the levels in those without. The HBV DNA serum level was inversely correlated to bacterial strains’ resistance to antimicrobials. However, further studies are warranted to understand the mechanisms behind this crosstalk.

## Additional Information

**How to cite this article**: Li, W. *et al.* Clinical correlation between HBV infection and concomitant bacterial infections. *Sci. Rep.* **5**, 15413; doi: 10.1038/srep15413 (2015).

## Figures and Tables

**Table 1:** Baseline information on patients with HBV infection co-infected by different bacteria.

	Patients (n = 491)
Sex	
Male, n (%)	395 (80)
Female, n (%)	96 (20)
Age [mean (range)]	47 (24–81)
Fibrosis stage, n (%)	
0–2	67 (14)
3	108 (22)
4	111 (23)
5	147 (30)
6	58 (12)
Liver cancer, n (%)	52 (11)
Years of infection [mean (range)]	11 (1–30)
Diabetes, n (%)	51 (10)
ALT (U/L)	94 (8–698)
Antiviral therapy n (%)	432 (88)
BMI, kg/m^2^	23 (16–39)

**Table 2:** Factors associated with HBV DNA serum concentration (log_10_ IU/mL) in patients with different co-infections.

Variable	*E. coli*(n = 268)		*S. aureus*(n = 61)		*P. aeruginosa*(n = 64)		*K. pneumoniae* (n = 98)	
	Uni	Multi	Uni	Multi	Uni	Multi	Uni	Multi
Age (years, continuous)	0.360		0.493		0.298		0.642	
Male sex	0.120		0.222		0.192		0.392	
Fibrosis (F1-F2 versus F3-F4)	0.030*	0.043*	0.069		0.098		0.022*	0.035*
Liver cancer (+/−)	0.020*	0.071	0.059		0.018*	0.032*	0.031*	0.048*
ALT (U/L, continuous)	0.029*	0.032*	0.008*	0.005*	0.010*	0.008*	0.028*	0.039*
GGT (U/L, continuous)	0.234		0.120		0.090		0.235	
BMI (kg/m^2^, continuous)	0.021*	0.009*	0.087		0.098		0.011*	0.021*
Platelets (/nL, continuous)	0.219		0.532		0.356		0.382	
Diabetes (+/−)	0.010*	0.083	0.032*	0.058	0.021*	0.045*	0.037*	0.061
HBeAg (+/−)	<0.001*	0.001*	0.002*	0.003*	0.003*	0.007*	0.003	0.009*
HBsAg (log_10_ IU/mL)	0.293		0.532		0.245		0.424	

^*^p < 0.05 compared with patients without HBV infections

**Table 3:** Comparison of drug resistance in isolates from patients with or without HBV infection infected with different bacteria.

	*E. coli*(P)	*P. aeruginosa* (P)	*K. pneumoniae* (P)		*S. aureus*(P)
Ampicillin	0.545	0.685	0.522	Amoxicillin	0.120
Ampicillin-sulbactam	0.033*	0.094	0.048*	Ciprofloxacin	0.010*
Amoxicillin-clavulanate	0.032*	0.043*	0.023*	Clindamycin	0.045*
Piperacillin	0.156	0.099	0.197	Gentamicin	0.043*
Piperacillin-tazobactam	0.198	0.032*	0.030*	Linezolid	0.121
Ceftizoxime	0.044*	0.033*	0.039*	Oxacillin	0.049*
Ceftazidime	0.021*	0.071	0.044*	Penicillin	0.694
Cefotaxime	0.024*	0.037*	0.043*	Dalfopristin	0.078
Cefepime	0.017*	0.015*	0.022*	Rifampicin	0.022*
Aztreonam	0.009**	0.009**	0.012*	Tetracycline	0.039*
Imipenem	0.129	0.283	0.211	Trimethoprim-sulfamethoxazole	0.021*
Meropenem	0.382	0.432	0.321	Ampicillin	0.058
Gentamicin	0.004**	0.009**	0.018*	Vancomycin	0.011*
Amikacin	0.010*	0.082	0.073	Erythromycin	0.015*
Ciprofloxacin	0.012*	0.031*	0.028*	Amikacin	0.384
Levofloxacin	0.006**	0.049*	0.023*	Furadantin	0.133
Tetracycline	0.011*	0.028*	0.042*	Tobramycin	0.029*
Chloramphenicol	0.098	0.084	0.111	Teicoplanin	0.071
Trimethoprim-sulfamethoxazole	0.012*	0.012*	0.016*	Trimethoprim	0.029*
ESBL	0.004**	0.009**	0.007**		

ESBL: extended spectrum β-lactamase; *p < 0.05 compared with patients without HBV infections; **p < 0.01 compared with patients without HBV infections.
